# Structural Characteristics of the Guaiacyl-Rich Lignins From Rice (*Oryza sativa* L.) Husks and Straw

**DOI:** 10.3389/fpls.2021.640475

**Published:** 2021-02-19

**Authors:** Mario J. Rosado, Jorge Rencoret, Gisela Marques, Ana Gutiérrez, José C. del Río

**Affiliations:** Department of Plant Biotechnology, Instituto de Recursos Naturales y Agrobiología de Sevilla, CSIC, Seville, Spain

**Keywords:** grasses, lignin, *p*-coumarates, ferulates, tricin, sinapyl *p*-coumarates

## Abstract

Rice (*Oryza sativa* L.) is a major cereal crop used for human nutrition worldwide. Harvesting and processing of rice generates huge amounts of lignocellulosic by-products such as rice husks and straw, which present important lignin contents that can be used to produce chemicals and materials. In this work, the structural characteristics of the lignins from rice husks and straw have been studied in detail. For this, whole cell walls of rice husks and straw and their isolated lignin preparations were thoroughly analyzed by an array of analytical techniques, including pyrolysis coupled to gas chromatography-mass spectrometry (Py-GC/MS), nuclear magnetic resonance (NMR), and derivatization followed by reductive cleavage (DFRC). The analyses revealed that both lignins, particularly the lignin from rice husks, were highly enriched in guaiacyl (G) units, and depleted in *p*-hydroxyphenyl (H) and syringyl (S) units, with H:G:S compositions of 7:81:12 (for rice husks) and 5:71:24 (for rice straw). These compositions were reflected in the relative abundances of the different interunit linkages. Hence, the lignin from rice husks were depleted in β–*O*–4′ alkyl-aryl ether units (representing 65% of all inter-unit linkages), but presented important amounts of β–5′ (phenylcoumarans, 23%) and other condensed units. On the other hand, the lignin from rice straw presented higher levels of β–*O*–4′ alkyl-aryl ethers (78%) but lower levels of phenylcoumarans (β–5′, 12%) and other condensed linkages, consistent with a lignin with a slightly higher S/G ratio. In addition, both lignins were partially acylated at the γ-OH of the side-chain (ca. 10–12% acylation degree) with *p*-coumarates, which overwhelmingly occurred over S-units. Finally, important amounts of the flavone tricin were also found incorporated into these lignins, being particularly abundant in the lignin of rice straw.

## Introduction

Lignin is a complex aromatic heteropolymer present in the cell-walls of vascular plants where it provides structural support, waterproofs the cell wall enabling transport of water and solutes through the vascular system, and acts as a barrier against pathogens. Unlike other natural biopolymers present in the plant cell wall (i.e., hemicelluloses, cellulose, proteins, etc.), that have a fixed and established structure, the structure of the lignin polymer lacks any regular order of repeating units and its composition widely fluctuate among taxa, from plant to plant, among different tissues of the same plant, and also with growing stage (Campbell and Sederoff, 1996; Donaldson, 2001; Vermerris and Boon, 2001; Rencoret et al., 2011; Lourenço et al., 2016). The high variability in lignin composition in plants is a consequence of the timing of the supply of the individual monomers to the lignifying zone and to the mechanism of lignin biosynthesis. Lignin is formed by the combinatorial oxidative radical coupling of three main monolignols, *p*-coumaryl, coniferyl, and sinapyl alcohols, that form the respective *p*-hydroxyphenyl (H), guaiacyl (G), and syringyl (S) lignin units when incorporated into the polymer, and this mechanism generates a series of substructures with a considerable variety of inter-unit linkages (β–*O*–4′, β–5′, β–β′, β–1′, 5–5′, 4–*O*–5′, etc.) within the polymer (Ralph et al., 2004; Vanholme et al., 2010, 2019). During the last few years, other phenolic compounds derived from beyond the canonical monolignol biosynthetic pathway have also been identified to behave as true lignin monomers participating in coupling and cross-coupling reactions with monolignols and being integrally incorporated into the lignin polymer (del Río et al., 2020). This is the case of the flavone tricin, that was found incorporated into the lignin structure in grasses and other monocots (del Río et al., 2012b; Rencoret et al., 2013; Lan et al., 2015, 2016a, b), or the hydroxystilbenes, particularly piceatannol, that were found incorporated into the lignins of palm fruit endocarps (del Río et al., 2017; Rencoret et al., 2018). The discoveries of these “novel” lignin monomers widely expanded our understanding of the lignin structure and revealed the structural complexity, heterogeneity, and variability of the lignin polymer.

Lignin is the only natural, high-molecular-weight polymer, with an aromatic backbone, making it an exceptional source for producing chemicals, biofuels, and materials that are currently obtained from fossil resources. Lignin is available in high amounts from lignocellulosic residues from the processing of agricultural or forest biomass. In this context, harvesting and processing of cereal crops, which are among the world’s most cultivated staple food crops, generate vast amounts of lignocellulosic by-products that can be used as low cost feedstocks to obtain lignin. Among them, rice (*Oryza sativa* L.), a perennial monocotyledonous grass belonging to the Poaceae, is one of the most cultivated and consumed cereals in the world. In 2018 rice paddy accounted for up to 167 million cultivated ha. with a global rice production of 782 million Mt (FAOSTAT, 2020). Harvesting and processing of rice generates huge amounts of two main by-products, namely rice husks and rice straw. The global production of these by-products amounted up to approximately 156 million Mt of husks (Gao et al., 2018), and over 730 million Mt of rice straw (Swain et al., 2019). These by-products are usually used as fodder or burned for co-generation of heat and power with the subsequent environmental problems (Kumar et al., 2016). However, rice husks and straw are lignocellulosic materials with important amounts of carbohydrates and lignin, and because their relatively low price and high availability, they have been considered excellent feedstocks for the production of chemicals, biofuels and bio-based materials (Lu and Hsieh, 2012; Kalita et al., 2015; Abraham et al., 2016; Gou et al., 2018; Swain et al., 2019; Sharma et al., 2020; Bhattacharyya et al., 2020).

As the lignin composition varies among different tissues of the same plant, it is expected that the lignins from rice husks and straw may have different compositions, a feature that can hinder the development of efficient conversion technologies for these lignocellulosic materials. Therefore, is imperative to know in detail the composition and structure of the lignins of these lignocellulosic materials for their efficient utilization. There have been few studies describing the lignin extraction from rice husks and straw after acidic and/or basic pretreatments although with limited attention paid to their composition (Kumar et al., 2016, 2019; Dagnino et al., 2018; Yeframova et al., 2019). However, studies regarding the detailed composition and the structural characteristics of the native lignins in rice husks and straw have been comparatively scarce. A previous work on the lignin from rice husks indicated that it was mainly formed by G- and H-lignin units, with minor amounts of S-units, and found evidences for β-*O*-4 alkyl-aryl ether, phenylcoumaran, and resinol substructures, but did not provide any additional structural information (Salanti et al., 2010). Other studies of the lignin in rice culms reported, besides the typical lignin inter-unit linkages (β-*O*-4, β-5, and β-β), the occurrence of *p*-coumaroylated lignin units and tricin (Lam et al., 2019; Takeda et al., 2019). In this article, we report the comprehensive structural characterization of the lignins of rice husks and straw by the use of different analytical techniques, including analytical pyrolysis coupled to gas chromatography and mass spectrometry (Py-GC/MS), two-dimensional nuclear magnetic resonance (2D-NMR), and the so-called derivatization followed by reductive cleavage (DFRC) degradation method. The lignin in the whole cell walls of rice husks and straw were first analyzed “*in situ*” by these analytical techniques, which provided information of the lignin characteristics without the need of their isolation, thus avoiding possible structural modifications during the isolation process. Then, for a more detailed structural characterization, the lignins from rice husks and straw were isolated by traditional procedures (Björkman, 1956), and subsequently analyzed by the same techniques. The results presented here will significantly improve our knowledge of the lignins from these important rice by-products that will help maximizing the industrial use of these materials, as well as providing important inputs for further bioengineering of cell wall lignin to improve the utilization of the rice biomass.

## Materials and Methods

### Rice Husks and Rice Straw Samples and Determination of Their Main Constituents

Samples of rice (*O. sativa* L., var. Indica, Puntal) husks and straw were obtained from a paddy field located in Isla Mayor (Seville, South Spain). The samples were air-dried and knife-milled using an IKA knife mill (Janke & Kunkel, Staufen, Germany) with 1 mm screen. The contents of extractives (acetone, methanol, and water–soluble extractives) were determined by successive extraction with acetone in a Soxhlet apparatus for 8 h, then with methanol (8 h), and finally with distilled water (8 h). The extractives contents were then determined gravimetrically after evaporating the solvents in a rotary evaporator. Klason lignin content was estimated as the residue after sulfuric acid hydrolysis of the pre-extracted material according to Tappi test method T222 om-88 (Tappi Standard Test Methods 2004-2005, 2004). The Klason lignin content was then corrected for proteins, determined from the N content measured in a LECO CHNS-932 Elemental Analyzer (LECO Corp., St. Joseph Mich.) using a 6.25 factor (Darwill et al., 1980), and ash (determined as indicated below for the whole samples). The acid-soluble lignin was determined, after the insoluble lignin was filtered off, by UV-spectroscopy at 205 nm using 110 L cm^–1^ g^–1^ as extinction coefficient, according to Tappi method UM 250 (Tappi Standard Test Methods 2004-2005, 2004). The holocellulose (hemicelluloses and α-cellulose) was isolated from the pre-extracted samples by delignification for 4 h using the acid chlorite method (Browning, 1967). The α-cellulose content was determined by removing the hemicelluloses from the holocellulose by alkali extraction (Browning, 1967). Finally, the ash content was determined by heating the samples for 6 h at 575°C in a muffle furnace. Three replicates were used for each sample.

### Isolation of “Milled-Wood Lignins” From Rice Husks and Straw

The “Milled-Wood Lignin” (MWL) preparations were isolated from rice husks and straw using the standard procedure (Björkman, 1956). Briefly, around 70 g of previously pre-extracted samples were finely milled using a Retsch PM100 planetary ball mill (Restch, Haan, Germany) for 5 h at 400 rpm using a 500 mL agate jar and agate ball bearings (20 × 20 mm). The milled samples were then extracted (3 × 12 h) with dioxane-water (90:10, v/v) (20 mL of solvent per gram of milled sample) and the isolated crude MWLs were subsequently purified as described elsewhere (del Río et al., 2012a). The isolated MWLs yields were ∼20% of the Klason lignin contents of the original material.

### Pyrolysis Coupled to Gas Chromatography and Mass Spectrometry

Pyrolysis of the whole cell walls of rice husks and straw and of their isolated MWLs (ca. 1 mg) were performed at 500°C in an EGA/PY-3030D microfurnace pyrolyzer (Frontier Laboratories Ltd., Fukushima, Japan) connected to a GC 7820A (Agilent Technologies, Inc., Santa Clara, CA, United States) equipped with a DB-1701 fused-silica capillary column (30 m × 0.25 mm i.d., 0.25 μm film thickness) and an Agilent 5975 mass-selective detector (EI at 70 EV). The oven temperature was programmed from 50° to 100°C at 20°C min^–1^ and then ramped to 280°C at a heating rate of 6°C min^–1^ and held for 5 min. The carrier gas was helium at 1 mL min^–1^. For the pyrolysis in the presence of tetramethylammonium hydroxide (TMAH), around 1 mg of sample were mixed with 0.5 mL of TMAH (25% w/w, in methanol), and the pyrolysis was carried out as described above. The released compounds were identified by comparison of their mass spectra with those of the Wiley and NIST libraries, with those reported in the literature (Ralph and Hatfield, 1991), and when possible, by comparison with the retention times and mass spectra of our own collection of authentic standards. Molar peak areas were calculated for the released pyrolysis products, the summed areas were normalized, and the data for two repetitive analyses were averaged and expressed as percentages. The relative standard deviation for the pyrolysis data was below 10%. No attempt was made to calculate the response factor for every single compound released. However, for most of the lignin-derived phenols, the response factors are quite similar (Bocchini et al., 1997), with the exception of vanillin, but this is a minor peak here.

### Two-Dimensional Nuclear Magnetic Resonance Spectroscopy

Two-dimensional nuclear magnetic resonance (2D-NMR) spectra were recorded on an AVANCE III 500 MHz instrument (Bruker, Karlsruhe, Germany) at the NMR facilities of the General Research Services of the University of Seville. For 2D-NMR of the whole cell walls, around 60 mg of finely ball-milled extractives-free samples were swollen in 0.6 mL of DMSO-*d*_6_ according to the method previously described (Kim et al., 2008; Rencoret et al., 2009). In the case of the MWLs, around 40 mg were dissolved in 0.5 mL of DMSO-*d*_6_. Heteronuclear Single Quantum Coherence (HSQC) experiments used Bruker’s standard “hsqcetgpsisp2.2” pulse program (adiabatic-pulsed version) using the parameters already described (del Río et al., 2012b). The central solvent peak was used as an internal reference (δ_*C*_ 39.5; δ_*H*_ 2.49). Signal assignments were made by comparison with literature (del Río et al., 2008, 2012b; Ralph et al., 2009; Rencoret et al., 2018). A semi-quantitative analysis of the volume integrals of the HSQC cross-relation signals was performed using Bruker’s Topspin 3.5 as previously described (del Río et al., 2012b). In the aliphatic oxygenated region, the relative abundances of side-chains involved in the various inter-unit linkages were estimated by integration of the areas of the C_α_ /H_α_ correlations (signals A_α_/A′_α_, B_α_, C_α_, C′_α_, D_α_, F_α_). The relative abundances of cinnamyl alcohol end-groups (I) were determined by integration of the C_γ_/H_γ_ correlation signals (I_γ_), whereas the abundance of cinnamaldehyde end-groups (J) was determined by integrating the signal from the C_8_/H_8_ correlations (J_8_) and comparing with that of I_β_. In the aromatic/unsaturated region, the signals used to quantitate the relative abundances of the aromatic units were S_2,6_, G_2_, H_2,6_, T_6_, FA_2_, *p*CA_2,6_; as signals S_2,6_, H_2,6_, and *p*CA_2,6_ involve two proton-carbon pairs, their volume integrals were halved. The relative abundances of *p*CA, FA, and T were referred to as a percentage of the total lignin units (S + G + H = 100%).

### Derivatization Followed by Reductive Cleavage

The derivatization followed by reductive cleavage (DFRC) was performed according to the originally developed method (Lu and Ralph, 1997a, b, 1998) and the detailed explanation of the experimental procedure can be found elsewhere (del Río et al., 2012a). Briefly, around 5 mg of MWL were stirred for 2 h at 50°C with acetyl bromide in acetic acid, 8:92 (v/v) and then treated with powdered Zn (50 mg) for 40 min at room temperature. The lignin degradation products were then acetylated for 1 h in 1.1 mL of dichloromethane containing 0.2 mL of acetic anhydride and 0.2 mL of pyridine. In order to assess the presence of naturally acetylated lignin units, the DFRC method was slightly modified to use propionylating reagents instead of acetylating ones (so-called DFRC′), as previously described (Ralph and Lu, 1998; del Río et al., 2007b). The lignin degradation products released by DFRC and DFRC′ were analyzed in a GCMS-QP2010plus instrument (Shimadzu Co., Kyoto, Japan) using a capillary column (DB-5MS 30 m × 0.25 mm I.D., 0.25 μm film thickness). The oven temperature was heated from 140 (1 min) to 250°C at a rate of 3°C min^–1^, then ramped at 3°C min^–1^ to 280°C (1 min) and finally ramped at 20°C min^–1^ to 300°C, and maintaining the final temperature for 18 min. The injector temperature was set at 250°C while the transfer line was kept at 310°C. The carrier gas was helium (1 mL min^–1^ flow rate). The relative molar abundances of the released lignin degradation products were determined using the molecular weights of their respective acetylated or propionylated compounds.

## Results

### Main Constituents of Rice Husks and Straw

The relative abundances of the main constituents (water-soluble material, acetone extractives, methanol extractives, Klason lignin, acid-soluble lignin, hemicelluloses, cellulose, proteins, and ash) of the rice husks and straw selected for this study are shown in Table 1. The lignin content in rice husks amounted up to 22.5% (including Klason and acid-soluble lignin contents) and was significantly higher than the lignin content in rice straw, where it accounted for 13.5%. On the other hand, the rice straw presented a higher content of extractives (totaling 17.3%, including acetone, methanol, and water-soluble extractives) than rice husks (10.7%). Both residues presented similar contents of hemicelluloses (28.3% in rice husks and 27.8% in rice straw), and cellulose (27.2% in rice husks and 24.0% in rice straw), and a very low content of proteins (0.7–0.9%). Also, both residues presented a high content of ash (10.6% in rice husks and 16.5% in rice straw), which mostly corresponded to silica, as indicated by other authors (Chandrasekhar et al., 2003; Salanti et al., 2010).

**TABLE 1:** Abundance (in percentage) of the main constituents of the dry weight of rice (*Oryza sativa* L.) husks and straw.^*a*^

	Rice husk	Rice straw
Acetone extractives	4.3 ± 0.3	3.4 ± 0.1
Methanol extractives	1.9 ± 0.3	5.8 ± 0.3
Water-soluble material	4.5 ± 0.3	8.1 ± 0.2
Klason lignin^b^	20.0 ± 0.1	12.4 ± 0.2
Acid-soluble lignin	2.5 ± 0.0	1.1 ± 0.0
Hemicelluloses	28.3 ± 0.7	27.8 ± 0.2
α-Cellulose	27.2 ± 0.2	24.0 ± 0.6
Proteins	0.7 ± 0.1	0.9 ± 0.1
Ash	10.6 ± 0.2	16.5 ± 0.3

*^a^Average of three replicates.^b^Corrected for proteins and ash.*

### Lignin Composition as Obtained by Py-GC/MS

**FIGURE 1:**
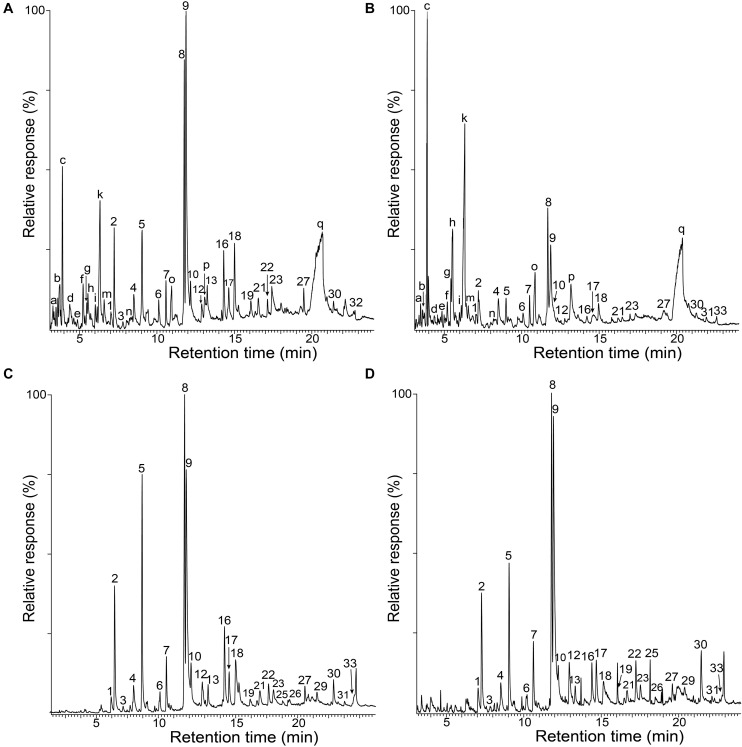
Py-GC/MS chromatograms of the whole cell walls of rice husks **(A)** and rice straw **(B)**, and of the isolated MWLs from rice husks **(C)** and rice straw **(D)**. The identities and relative abundances of the released phenolic compounds are listed in Table 2. Letters refer to carbohydrate-derived compounds: (a) (3*H*)-furan2-one; (b) (2*H*)-furan-3-one; (c) furfural; (d) 2-hydroxymethylfuran; (e) cyclopenten-1-ene-3,4-dione; (f) 2,3-dihydro-5-methylfuran-2-one; (g) 5-methyl-2-furfuraldehyde; (h) 3-ethyldihydro-2(*3H*)-furanone; (i) (5*H*)-furan-2-one; (k) 4-hydroxy-5,6-dihydro-(2*H*)-pyran-2-one; (m) 2-hydroxy-3-methyl- 2-cyclopenten-1-one; (n) 3-hydroxy-2-methyl-(4*H*)-pyran-4-one; (o) 5-hydroxymethyl-2-tetrahydrofuraldehyde-3-one; (p) 5-hydroxymethyl-2-furfuraldehyde; (q) levoglucosan.

The whole cell walls of rice husks and straw, and their isolated MWLs, were first analyzed by Py-GC/MS that provided useful information regarding the composition of the lignocellulosic materials. The pyrograms of the whole cell walls of rice husks (Figure 1A) and rice straw (Figure 1B) showed compounds released from both carbohydrates (peaks a–q) and lignin (peaks 1–33). The identities and relative molar abundances of the released lignin-derived phenolic compounds are listed in Table 2, whereas the identities of the carbohydrate-derived compounds are detailed in the legend of Figure 1. Interestingly, the pyrolysis of rice husks released higher amounts of lignin-derived compounds, whereas the pyrolysis of rice straw released higher amounts of carbohydrate-derived compounds, in agreement with the higher lignin content observed in rice husks (22.5%) compared to rice straw (13.5%), as shown in Table 1. In both cases, the main phenolic compounds released were 4-vinylguaiacol (8) and 4-vinylphenol (9). The pyrograms of the MWLs isolated from rice husks (Figure 1C) and rice straw (Figure 1D) only released phenolic compounds arising from lignin (and from *p*-hydroxycinnamates), that, in general terms, matched the profile of the phenolic compounds released from the corresponding whole cell walls (Figures 1A,B). The most abundant phenolic compounds released from both lignins were 4-vinylguaiacol (8) and 4-vinylphenol (9), as occurred in the pyrolysis of their respective whole cell walls. In addition, important amounts of phenolic compounds derived from guaiacyl (G)-lignin units, such as guaiacol (2), 4-methylguaiacol (5), and 4-ethylguaiacol (7), among others, were released from both lignins. Phenolic compounds derived from *p*-hydroxycinnamyl (H)-lignin units, such as phenol (1), 4-methylphenol (4), 4-ethylphenol (6), and from syringyl (S)-lignin units, such as syringol (12), 4-methylsyringol (17), 4-ethylsyringol (19), and 4-vinylsyringol (22), among others, were also released from both lignins, although in much lower amounts than their respective G-counterparts. In principle, the H:G:S composition of both lignins could be assessed from the relative abundances of the phenolic compounds derived from the different H, G, and S-lignin units. However, in the case of grasses, *p*-hydroxycinnamates (*p*-coumarates and ferulates) are also important part of the lignins, with *p*-coumarates acylating the lignin side-chains, and ferulates acylating the arabinosyl residues of arabinoxylans and also forming covalent linkages with the lignin core (Ralph, 2010; Hatfield et al., 2017). *p*-Hydroxycinnamates are known to decarboxylate upon pyrolysis producing the respective 4-vinylphenol (from *p*-coumarates) and 4-vinylguaiacol (from ferulates), which hinder the effective estimation of the lignin H:G:S composition (del Río et al., 1996, 2007a). A close estimation of the lignin H:G:S compositions were, however, obtained by ignoring the 4-vinylpyhenol (that can arise from both H-lignin and *p*-coumarates) and 4-vinylguaiacol (that can arise from G-lignin and from ferulates), as well as the respective 4-vinylsyringol, as previously and successfully done for other grasses (del Río et al., 2012a, b, 2015, 2018; Rencoret et al., 2015). The lignin composition thus estimated is shown in Table 2, and indicated that the lignin from rice husks presented a H:G:S composition of 13:73:14 (S/G ratio of 0.19) where as the lignin from rice straw presented a H:G:S composition of 16:61:23 (S/G ratio of 0.37). Therefore, the Py-GC/MS data indicated that both lignins were highly enriched in G-lignin units with the occurrence of lower amounts of H- and S-lignin units, with the lignin of rice husks being particularly highly enriched in G-units.

**TABLE 2:** Identities and relative molar abundances of the compounds released after Py-GC/MS of whole cell walls (CW) of rice (*Oryza sativa* L.) husks and straw and their respective isolated MWLs.

			Rice husks		Rice straw	
Label	Compound	Origin	CW	MWL	CW	MWL
1	Phenol	H	1.4	2.1	2.3	2.7
2	Guaiacol	G	6.4	8.8	5.1	8.0
3	3-methylphenol	H	0.8	0.6	0.9	0.6
4	4-methylphenol	H	1.8	3.1	3.5	3.4
5	4-methylguaiacol	G	8.3	13.1	5.9	8.3
6	4-ethylphenol	H	1.4	0.5	2.0	0.7
7	4-ethylguaiacol	G	2.5	2.9	4.6	3.8
8	4-vinylguaiacol	G/FA	12.4	15.4	19.6	14.5
9	4-vinylphenol	H/*p*CA	33.4	27.1	31.2	30.6
10	Eugenol	G	3.3	2.3	0.7	2.5
11	4-allylphenol	H	0.3	0.3	0.3	0.4
12	Syringol	S	1.0	1.8	2.1	3.5
13	*cis*-isoeugenol	G	2.2	1.1	0.6	1.0
14	*cis*-4-propenylphenol	H	0.2	0.2	0.2	0.2
15	*trans*-4-propenylphenol	H	0.6	0.6	1.1	0.5
16	*trans*-isoeugenol	G	4.3	4.7	2.6	2.6
17	4-methylsyringol	S	2.4	1.9	1.7	2.6
18	Vanillin	G	5.8	4.6	5.8	3.6
19	4-ethylsyringol	S	1.0	0.6	0.5	0.7
20	Vanillic acid methyl ester	G	0.3	0.4	0.2	0.4
21	Acetoguaiacone	G	0.9	0.9	0.8	0.7
22	4-vinylsyringol	S	0.9	1.2	0.3	2.2
23	Guaiacylacetone	G	2.8	1.3	2.2	1.1
24	4-allylsyringol	S	0.2	0.2	0.4	0.3
25	Propiovanillone	G	0.8	0.4	0.2	0.1
26	*cis*-4-propenylsyringol	S	0.2	0.2	0.5	0.3
27	*trans*-4-propenylsyringol	S	1.1	0.8	1.6	0.9
28	Vanillic acid	G	0.4	0.6	0.0	0.2
29	Syringaldehyde	S	0.6	0.7	0.6	0.8
30	Acetosyringone	S	0.5	1.3	1.4	2.5
31	Syringylacetone	S	0.3	0.3	1.0	0.3
32	*trans*-coniferaldehyde	G	1.5	0.0	0.0	0.0
33	Propiosyringone	S	0.1	0.1	0.3	0.2
	%H*=		12.2	13.0	21.0	16.2
	%G*=		74.2	73.0	58.4	61.0
	%S*=		13.6	14.0	20.6	22.8
	S/G ratio*=		0.18	0.19	0.35	0.37

*Calculated without taken into account the vinyl compounds 8, 9, and 22.H, p-hydroxyphenyl lignin units; G, guaiacyl lignin units; S, syringyl lignin units; pCA, p-coumarates; FA, ferulates.

**FIGURE 2:**
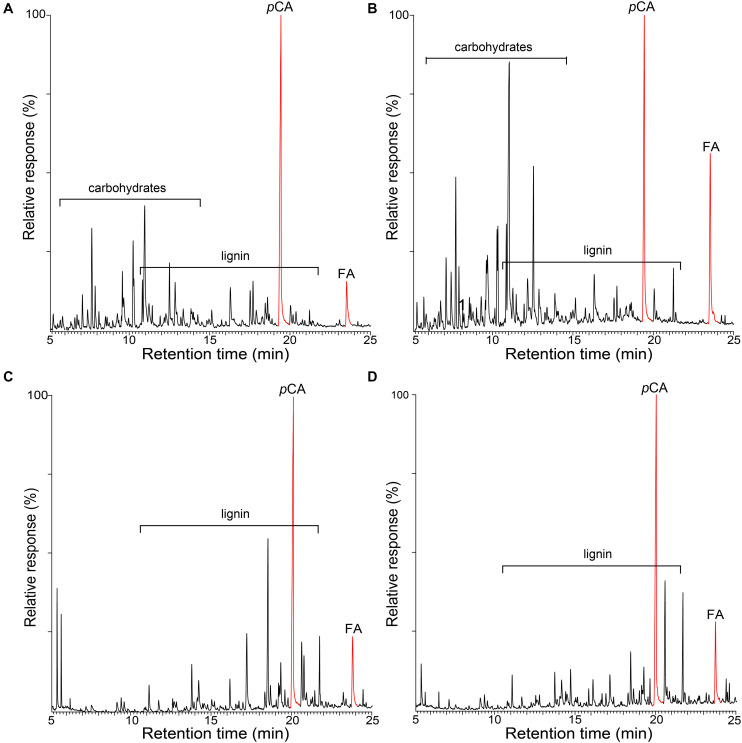
Py-TMAH-GC/MS chromatograms of the whole cell walls of rice husks **(A)** and rice straw **(B)**, and of the isolated MWLs from rice husks **(C)** and rice straw **(D)**. *p*CA is the fully methylated *p*-coumaric acid (methyl *trans*-4-*O*-methyl-*p*-coumarate); FA is the fully methylated ferulic acid (methyl *trans*-4-*O*-methyl-ferulate).

The occurrence of *p*-hydroxycinnamates (*p*-coumarates and ferulates) in these lignins was confirmed by performing the pyrolysis in the presence of a methylating reagent, TMAH. Pyrolysis in the presence of TMAH (Py-TMAH) prevents decarboxylation of *p*-hydroxycinnamates and releases them intact as their fully methylated derivatives (del Río et al., 1996, 2007a). The Py-TMAH pyrograms of the whole cell walls of rice husks and straw and of their respective isolated MWLs are shown in Figure 2, and clearly show the release of important amounts of the fully methylated derivatives of *p*-coumaric (methyl *trans*-4-*O*-methyl-*p*-coumarate, *p*CA) and ferulic (methyl 4-*O*-methyl-ferulate, FA) acids. Therefore, the Py-TMAH analysis conclusively demonstrated the presence of significant amounts of *p*-coumarates and ferulates in the lignins of rice husks and rice straw.

### Lignin Units and Inter-Unit Linkages as Seen by 2D-NMR

**FIGURE 3:**
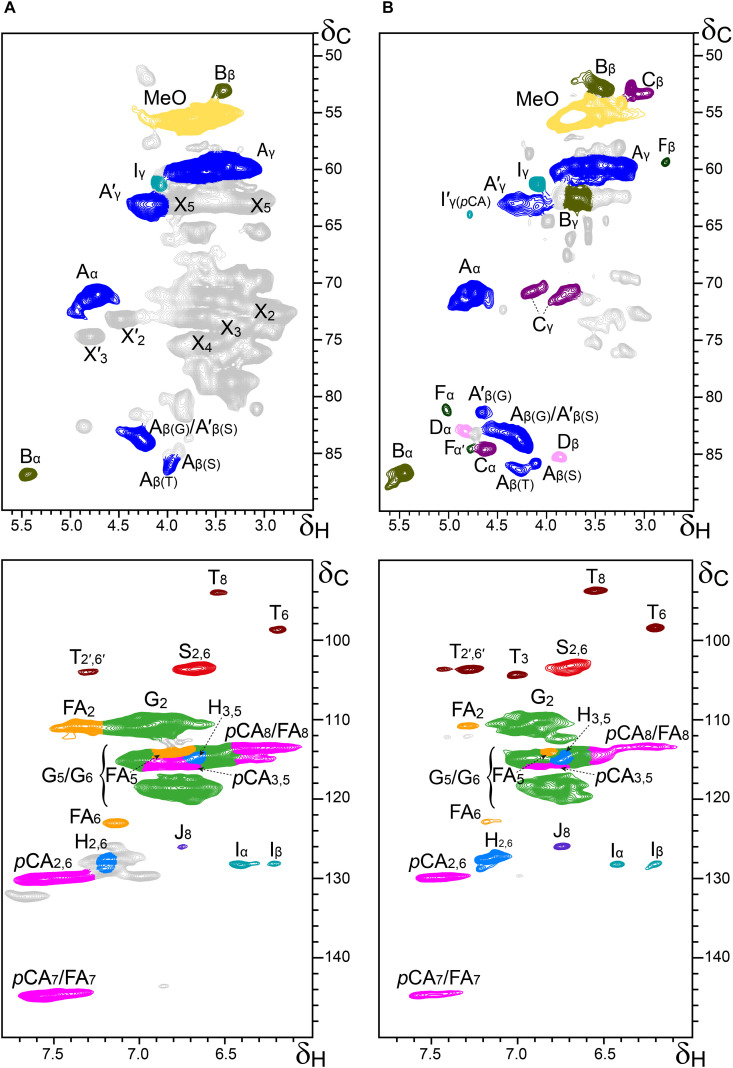
Aliphatic-oxygenated (δ_*C*_/δ_*H*_ 48–90/2.5–5.7; top) and aromatic (δ_*C*_/δ_*H*_ 90–150/6.0–7.8; bottom) regions from the 2D-HSQC-NMR spectra (in DMSO-*d*_6_) of the whole cell walls from rice husks **(A)** and its isolated MWL **(B)**. The main structures identified are drawn in Figure 5. See Table 3 for signal assignments.

**FIGURE 4:**
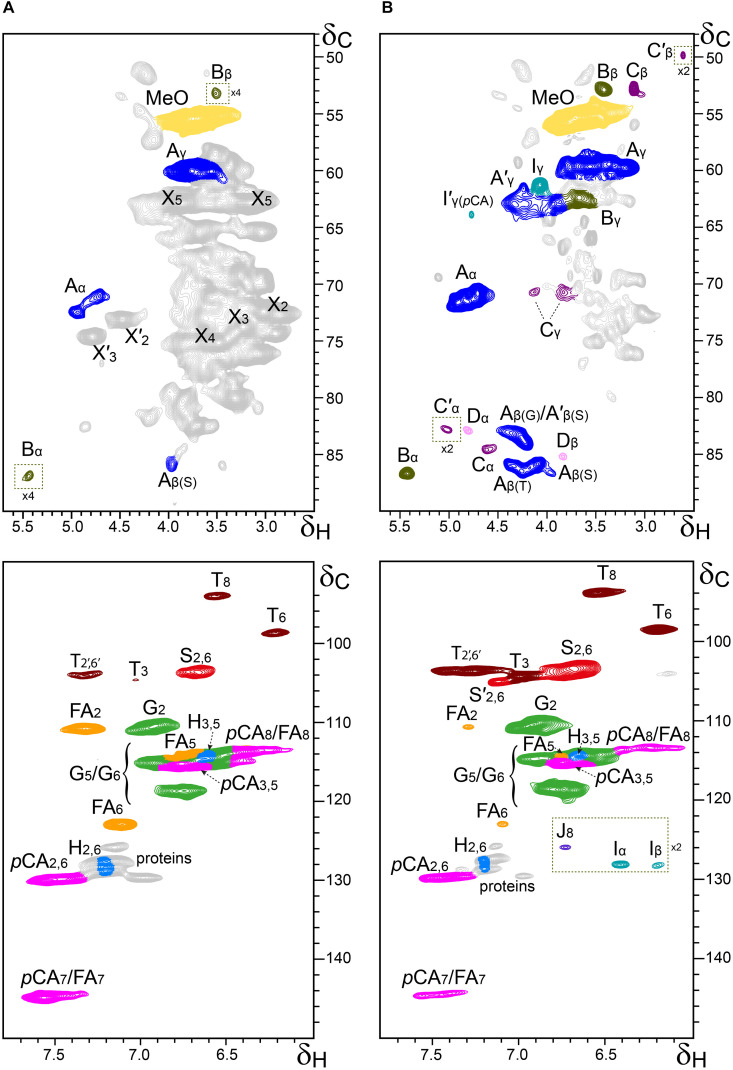
Aliphatic-oxygenated (δ_*C*_/δ_*H*_ 48–90/2.5–5.7; top) and aromatic (δ_*C*_/δ_*H*_ 90–150/6.0–7.8; bottom) regions from the 2D-HSQC-NMR spectra (in DMSO-*d*_6_) of the whole cell walls from rice straw **(A)** and its isolated MWL **(B)**. The main structures identified are drawn in Figure 5. See Table 3 for signal assignments.

**FIGURE 5:**
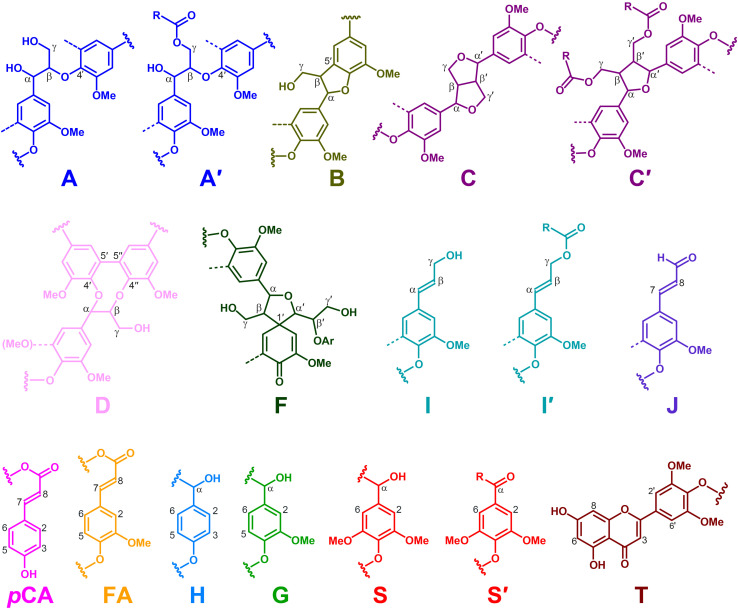
Main structures identified in the NMR spectra of the lignins of rice husks and straw. **A**, β–*O*–4′ alkyl-aryl ethers; **A′**, β–*O*–4′ alkyl-aryl ethers acylated at the γ-OH; **B**, phenylcoumarans; **C**, resinols; **C′**, tetrahydrofurans acylated at the γ-OH; **D**, dibenzodioxocins; **F**, spirodienones; **I**, *p*-hydroxycinnamyl alcohol end-groups; **I′**, *p*-hydroxycinnamyl alcohol end-groups acylated at the γ-OH; **J**, cinnamaldehyde end-groups; ***p*CA**, *p*-coumarates; **FA**, ferulates; **H**, *p*-hydroxyphenyl units; **G**, guaiacyl units; **S**, syringyl units; **S′**, Cα-oxidized S-units; **T**, tricin. The structures are colored in order to match the signals in the HSQC spectra of Figures 3, 4.

Additional information about the composition of the lignin units as well as the inter-unit linkages present in the lignins from rice husks and straw were obtained by 2D-NMR spectroscopy (in HSQC experiments). The side-chain (δ_*C*_/δ_*H*_ 48–90/2.5–5.7) and the aromatic/unsaturated (δ_*C*_/δ_*H*_ 90–150/6.0–7.8) regions of the HSQC spectra of the whole cell walls of rice husks and straw and of their isolated MWLs, are shown in Figures 3, 4. The spectra of the whole cell walls showed signals from carbohydrates and lignin, whereas the spectra of the isolated MWLs showed mostly signals from the lignin polymer, evidencing the efficiency of the lignin isolation process. The HSQC spectrum of the whole cell walls of rice husks presented higher intensities of the lignin signals than the spectrum of the whole cell walls of rice straw, as corresponded to their higher lignin content. The lignin correlation signals assigned in the HSQC spectra are listed in Table 3 and the main lignin units and substructures identified are depicted in Figure 5. Carbohydrate signals from the different correlations of xylans (X_2_, X_3_, X_4_, and X_5_), including acetylated xylans (X′_2_ and X′_3_), were predominant in the aliphatic side-chain region of the spectra of the whole cell walls, partially overlapping with some lignin signals. The spectra of the isolated MWLs, however, exhibited predominantly lignin signals that matched those present in the HSQC spectra of their respective whole cell walls indicating that the MWL preparations were representative of the native lignins.

**TABLE 3:** Assignments of the lignin ^1^H/^13^C correlation signals in the 2D HSQC spectra from the whole cell walls of rice husks and straw and their isolated MWLs (in DMSO–*d*_6_).

Label	δ_*C*_/δ_*H*_ (ppm)	Assignment
C′_β_	49.7/2.58	C_β_/H_β_ in γ-acylated β–β′ tetrahydrofurans (**C′**)
B_β_	53.1/3.41	C_β_/H_β_ in β–5′ phenylcoumarans (**B**)
C_β_	53.6/3.07	C_β_/H_β_ in β–β′ resinols (**C**)
−OCH_3_	55.6/3.73	C/H in methoxyls
A_γ_	59.4/3.20, 3.56	C_γ_/H_γ_ in normal (γ-hydroxylated) β–*O*–4′ substructures (**A**)
F_β_	59.3/2.76	C_β_/H_β_ in spirodienones (**F**)
I_γ_	61.5/4.09	C_γ_/H_γ_ in cinnamyl alcohol end–groups (**I**)
B_γ_	62.6/3.68	C_γ_/H_γ_ in β–5′ phenylcoumarans (**B**)
A′_γ_	62.7/3.83, 4.30	C_γ_/H_γ_ in γ-acylated β–*O*–4′ substructures (**A′**)
I′_γ (_*_*p*_*_*CA)*_	64.0/4.77	C_γ_/H_γ_ in γ-*p*-coumaroylated cinnamyl alcohol end–groups (**I′**)
C_γ_	71.0/3.82, 4.18	C_γ_/H_γ_ in β–β′ resinols (**C**)
A_α_	71.8/4.87	C_α_/H_α_ in β–*O*–4′ substructures (**A**)
A′_β(G)_	81.2/4.64	C_β_/H_β_ in γ-acylated β–*O*–4′ alkyl-aryl ethers (**A′**) linked to a G unit
F_α_	81.2/5.02	C_α_/H_α_ in spirodienones (**F**)
C′_α_	82.6/5.00	C_α_/H_α_ in γ-acylated β–β′ tetrahydrofurans (**C′**)
A′_β(S)_	83.0/4.33	C_β_/H_β_ in γ-acylated β–*O*–4′ alkyl-aryl ethers (**A′**) linked to a S unit
D_α_	83.3/4.82	C_α_/H_α_ in 5–5′ dibenzodioxocins (**D**)
A_β(G)_	83.9/4.28	C_β_/H_β_ in β–*O*–4′ alkyl-aryl ethers (**A**) linked to a G unit
C_α_	84.9/4.67	C_α_/H_α_ in β–β′ resinols (**C**)
F_α′_	85.2/4.72	C_α′_/H_α′_ in spirodienones (**F**)
D_β_	85.4/3.86	C_β_/H_β_ in 5–5′ dibenzodioxocins (**D**)
A_β(S)_	85.9/4.12	C_β_/H_β_ in β–*O*–4′ alkyl-aryl ethers **(A)** linked to a S unit
A_β(T)_	86.1/4.31	C_β_/H_β_ in β–*O*–4′ substructures (**A**) linked to tricin
B_α_	86.9/5.47	C_α_/H_α_ in phenylcoumarans (**B**)
T_8_	94.0/6.56	C_8_/H_8_ in tricin (**T**)
T_6_	98.7/6.22	C_6_/H_6_ in tricin (**T**)
S_2_,_6_	103.8/6.69	C_2_/H_2_ and C_6_/H_6_ in etherified syringyl units (**S**)
T_2′,6′_	103.9/7.28	C_2′_/H_2′_ and C_6′_/H_6′_ in tricin (**T**)
T_3_	104.6/7.02	C_3_/H_3_ in tricin (**T**)
G_2_	110.9/7.00	C_2_/H_2_ in guaiacyl units (**G**)
FA_2_	111.0/7.32	C_2_/H_2_ in ferulates (**FA**)
*p*CA_8_/FA_8_	113.5/6.30	C_8_/H_8_ in *p*–coumarates (***p*CA**) and ferulates (**FA**)
H_3,5_	114.5/6.68	C_3_/H_3_ and C_5_/H_5_ in *p*-hydroxyphenyl units (**H**)
G_5_/G_6_	114.9/6.74, 6.94	C_5_/H_5_ and C_6_/H_6_ in guaiacyl units (**G**)
FA_5_	115.4/6.88	C_5_/H_5_ in ferulates (**FA**)
*p*CA_3,5_	115.5/6.77	C_3_/H_3_ and C_5_/H_5_ in *p*-coumarates (***p*CA**)
FA_6_	123.3/7.10	C_6_/H_6_ in ferulates (**FA**)
J_8_	126.3/6.76	C_8_/H_8_ in cinnamaldehyde end-groups (**J**)
H_2,6_	127.6/7.17	C_2_/H_2_ and C_6_/H_6_ in *p*-hydroxyphenyl units (**H**)
I_β_	128.2/6.21	C_β_/H_β_ in cinnamyl alcohol end–groups (**I**)
I_α_	128.4/6.44	C_α_/H_α_ in cinnamyl alcohol end–groups (**I**)
*p*CA_2,6_	129.9/7.45	C_2_/H_2_ and C_6_/H_6_ in *p*-coumarates (***p*CA**)
*p*CA_7_/FA_7_	144.4/7.41	C_7_/H_7_ in *p*–coumarates (***p*CA**) and ferulates (**FA**)

In the aliphatic-oxygenated region of the spectra, besides the signal from methoxyls, the rest of signals corresponded to the different lignin inter-unit linkages. Typical signals from the C_α_/H_α_, C_β_/H_β_, and C_γ_/H_γ_ correlations of β–*O*–4′ alkyl-aryl ethers (**A**), phenylcoumarans (**B**), resinols (**C**), dibenzodioxocins (**D**), spirodienones (**F**), and cinnamyl alcohol end-groups (**I**), were observed in this region of the spectra. The occurrence of strong signals from condensed lignin structures, such as phenylcoumarans (**B**), and particularly from dibenzodioxocins (**D**), which essentially involved G-lignin units linked by β–5 and 5–5 bonds, respectively, was indicative of the enrichment of G-lignin units in these lignins. An important feature observed in the HSQC spectra of these lignins was the occurrence of strong signals from γ-acylated lignin structures, mainly from γ-acylated β–*O*–4′ alkyl aryl ethers (**A′**). The occurrence of intense signals at around δ_*C*_/δ_*H*_ 62.7/3.83-4.30, assigned to the C_γ_/H_γ_ correlations of γ-acylated β–*O*–4′ substructures (A′_γ_), revealed that a significant part of the lignins from rice husks and straw were acylated at the γ-OH of the lignin side-chain. Signals for the C_β_/H_β_ correlations of γ-acylated β–*O*–4′ substructures linked to S-units (A′_β(S)_) overlapped with those of normal γ-OH β–*O*–4′ substructures linked to G-units (A_β(G)_) at around δ_*C*_/δ_*H*_ 83.0/4.33, whereas the signal for the C_β_/H_β_ correlations of γ-acylated β–*O*–4′ substructures linked to G-units (A′_β(G)_) were clearly observed at around δ_*C*_/δ_*H*_ 80.5/4.53. The extent of γ-acylation of these lignins were estimated from the C_γ_/H_γ_ correlation signals of normal γ-OH (A_γ_) and γ-acylated β–*O*–4′ substructures (A′_γ_) in the HSQC spectra of the isolated MWLs, where the signals from carbohydrates do not interfere, and revealed that 10% of the lignin side-chains of rice husks, and 12% of the lignin side-chains of rice straw were acylated at the γ-OH. Likewise, signals for the C_α_/H_α_ and C_β_/H_β_ correlations of γ-acylated tetrahydrofuran structures arising from β–β-coupling of two γ-acylated monolignols (**C′**) could be observed in the spectrum of the lignin from rice straw at δ_*C*_/δ_*H*_ 82.6/5.00 (C′_α_) and 49.7/2.58 (C′_β_), although at lower intensities, providing additional evidences of the partial acylation of the lignin side-chain. In addition, a signal for the C_γ_/H_γ_ correlations of cinnamyl alcohol end-groups acylated at the γ-OH of the lignin side-chain (**I′**) was also observed at δ_*C*_/δ_*H*_ 64.0/4.77 (I′_γ(_*_*p*_*_*CA)*_). This signal is clearly distinctive of γ-acylation with *p*-coumarates, and is different from the signals of γ-acylation with other groups, such as acetates that should appear at δ_*C*_/δ_*H*_ 64.0/4.65, or *p*-hydroxybenzoates that should appear at δ_*C*_/δ_*H*_ 64.4/4.87 (Rencoret et al., 2018). Therefore, this signal clearly evidenced that the cinnamyl alcohol end-groups were partially acylated with *p*-coumarates, which may also be the main group acylating these lignins.

In the aromatic/unsaturated region of the HSQC spectra, the main correlation signals corresponded to the aromatic rings of the different lignin units (**H**, **G**, and **S**), including Cα-oxidized S-lignin units (**S′**), as well as to the aromatic rings and the unsaturated side-chains of *p*-coumarates (***p*CA**) and ferulates (**FA**). The signals for H-lignin units were only observed at low intensities, and in some cases overlapped with signals from proteins. Other signals in the aromatic/unsaturated region of the spectra were from the C_α_/H_α_ and C_β_/H_β_ correlations of cinnamyl alcohol end-groups (**I**) and for the C_8_/H_8_ correlations of cinnamaldehydes (**J**). In addition, in this region of the spectra also appeared the two distinctive signals corresponding to the C_8_/H_8_ and C_6_/H_6_ correlations of tricin (**T**), together with the signals for their C_3_/H_3_ and C_2′,6′_/H_2′,6′_ correlations (del Río et al., 2012b).

The structural characteristics of the lignins from rice husks and straw, such as the relative abundances of the different interunit linkages, β–*O*–4′ aryl ethers (**A**/**A**′), β–5′ phenylcoumarans (**B**), β–β′ resinols (**C**), β–β′ tetrahydrofurans (**C′**), 5–5′ dibenzodioxocins (**D**), β–1′ spirodienones (**F**), and cinnamyl alcohol (**I**), and cinnamaldehyde (**J**) end-groups, the percentage of γ-acylation of the lignin side-chain, the relative abundances of the lignin units (**H**, **G**, and **S**), *p*-coumarates (***p*CA**), ferulates (**FA**), and tricin (**T**), estimated from volume integration of the signals in the HSQC spectra, are indicated in Table 4. The 2D-NMR data confirmed that the lignins from rice husks and straw were enriched in G-lignin units and depleted in H- and S-lignin units, as already revealed by Py-GC/MS. The H:G:S composition of the lignins from rice husks (7:81:12; S/G of 0.15) and rice straw (5:71:24; S/G of 0.34) basically matched those obtained upon Py-GC/MS, and indicated that the lignin from rice husks was particularly highly enriched in G-units. The 2D-NMR data also indicated that *p*-coumarates and ferulates were important components in the lignins from rice husks and straw, as already shown by Py-TMAH. Interestingly, ferulates were present in lower abundance in the isolated MWLs than in the respective whole cell walls, which was reflected in the *p*CA/FA ratio, that was lower in the whole cell walls of (1.5 and 0.7, in rice husks and straw) than in their respective isolated MWLs (3.0 and 4.0, in the MWLs of rice husks and straw). This indicated that ferulates were predominantly attached to the carbohydrates, which were removed during the MWL isolation process, whereas *p*-coumarates were mostly linked to the lignin structure. The occurrence of important amounts of *p*-coumarates in these lignins is an indication that they might be the groups acylating the γ-OH of the lignin side-chain, a typical feature of grass lignins (Ralph, 2010). Finally, the flavone tricin was also present in both lignins in significant amounts, being more abundant in the lignin from rice straw (27% referred to total lignin units) than in the lignin from rice husks (7%). However, it is important to note that the quantitation of tricin by 2D-NMR (as well as of *p*-coumarates and ferulates) is overestimated due to the longer relaxation time of these end-units. The differences in the composition between the lignins from rice husks and straw, with the former being highly enriched in G-lignin units, were reflected in the relative abundances of the various interunit linkages, as shown in Table 4. Hence, the lignin from rice husks presented lower levels of β–*O*–4 alkyl aryl ether structures (65% of all measured linkages) and higher levels of condensed structures such as phenylcoumarans (23%), as corresponds to a lignin highly enriched in G-lignin units, together with minor amounts of other condensed structures (dibenzodioxocins, 5%; resinols, 4%; spirodienones, 3%), as well as cinnamyl alcohol (6%) and cinnamaldehyde (5%) end-groups. On the other hand, the lignin from rice straw, with a slightly higher S/G ratio, presented a higher level of β–*O*–4 alkyl aryl ether structures (78%), and lower levels of phenylcoumarans (12%), together with minor amounts of other condensed structures (dibenzodioxocins, 4%; resinols, 4%; tetrahydrofurans, 1%; spirodienones, 1%) and cinnamyl alcohol (6%) and cinnamaldehyde (5%) end-groups.

**TABLE 4:** Structural characteristics (lignin inter-unit linkage types, end-groups, γ-acylation, aromatic units, and S/G ratio, cinnamate content, and tricin content) from integration of ^1^H/^13^C correlation signals in the HSQC spectra of the whole cell walls (CW) of rice husks and rice straw and their isolated MWLs.

	Rice husks		Rice straw	
	CW	MWL	CW	MWL
**Lignin inter-unit linkages (%)**				
β–*O*–4′ aryl ethers (**A**/**A′**)	–	65	–	78
β–5′ Phenylcoumarans (**B**)	–	23	–	12
β–β′ Resinols (**C**)	–	4	–	4
β–β′ Tetrahydrofurans (**C′**)	–	0	–	1
5–5′ Dibenzodioxocins (**D**)	–	5	–	4
β–1′ Spirodienones (**F**)	–	3	–	1
**Lignin end-groups^*a*^**				
Cinnamyl alcohol end-groups (**I**)	–	6	–	6
Cinnamaldehyde end-groups (**J**)	–	5	–	5
**Lignin side-chain γ-acylation (%)**	–	10	–	12
**Lignin aromatic units^*b*^**				
**H** (%)	5	7	7	5
**G** (%)	85	81	71	71
**S** (%)	10	12	22	24
**S**/**G** ratio	0.12	0.15	0.31	0.34
***p*-Hydroxycinnamates^*c*^**				
*p*-coumarates (***p*CA**)	28	12	39	16
ferulates (**FA**)	19	4	53	4
***p*CA**/**FA** ratio	1.5	3.0	0.7	4.0
Tricin (**T**)^*c*^	5	7	18	27

*^a^Expressed as a fraction of the total lignin inter-unit linkage types **A–F**.^b^Relative percentages (H + G + S = 100).^c^pCA, FA, and T molar contents as percentages of total lignin content (H + G + S = 100).*

### Nature of Lignin Acylation as Seen by DFRC

The HSQC spectra of the lignins of rice husks and straw revealed that they were partially acylated (10–12%) at the γ-OH of the lignin side-chains but did not provide information regarding the nature of the acylating groups. To assess the nature of the acyl groups present at the γ-OH of the lignin side-chain, the MWLs were analyzed by DFRC, a chemical degradative method that cleaves β-ether bonds, the most abundant linkages in the lignin structure, releasing the lignin units that are involved in those bonds. A characteristic feature of this degradation method is that it maintains intact the ester bonds present at the γ-OH of the lignin side chain, and thus can provide important information about the nature of the groups acylating the γ-OH.

**FIGURE 6:**
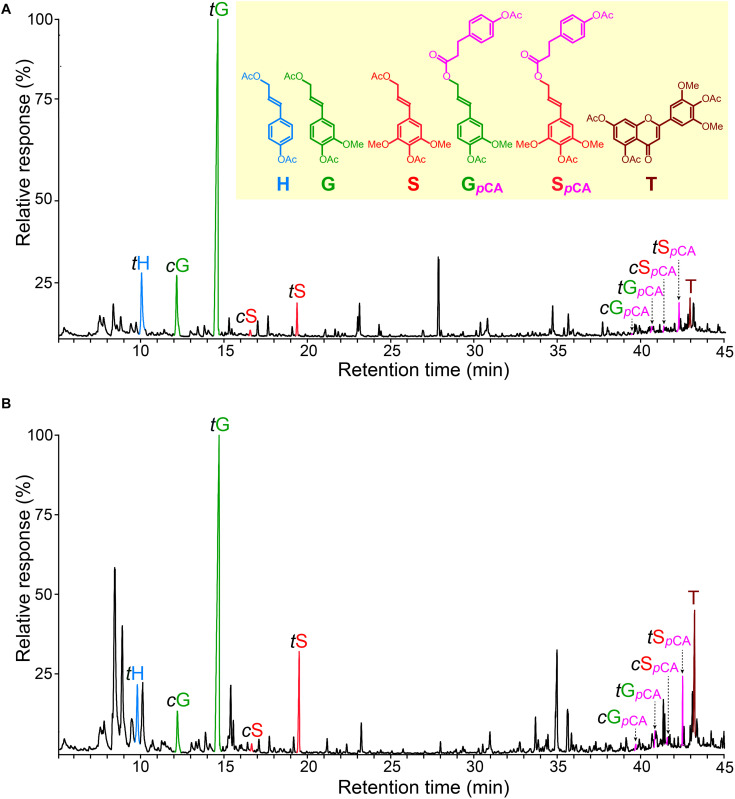
Chromatograms of the DFRC degradation products released from the MWLs isolated from rice husk **(A)** and rice straw **(B)** showing the presence of sinapyl (and minor coniferyl) units acylated by *p*-coumarate moieties. *t*H, *c*G, *t*G, *c*S, and *t*S are the normal *cis*- and *trans-p*-hydroxyphenyl (H), coniferyl (G), and sinapyl (S) alcohol monomers (as their acetate derivates); *c*G_*pCA*_, *t*G_*pCA*_, *c*S_*pCA*_, and *t*S_*pCA*_ are the *cis*- and *trans*-coniferyl and sinapyl dihydro-*p*-coumarates (as their acetate derivatives); T is tricin (as its acetate derivative).

The chromatograms of the DFRC degradation products released from the lignins of rice husks and straw (Figure 6) showed the occurrence of the *cis*- and *trans*-isomers of the *p*-hydroxyphenyl (*t*H), guaiacyl (*c*G and *t*G), and syringyl (*c*S and *t*S) lignin monomers (as their acetate derivatives) arising from normal γ-OH lignin units involved in β-ether linkages. But more important, the chromatograms also showed the release of the *cis*- and *trans*-isomers of S-lignin units acylated with *p*-coumarates (*c*S_*pCA*_ and *t*S_*pCA*_), as well as minor amounts of the guaiacyl analogs (*c*G_*pCA*_ and *t*G_*pCA*_). The release of these compounds confirmed that *p*-coumarates were the groups partially acylating the γ-OH of these lignins, and that *p*-coumaroylation preferentially occurred over S units. On the other hand, the lignins from many plants, including other grasses, also present acetate groups acylating the γ-OH of the lignin side-chain (Ralph, 1996; Ralph and Lu, 1998; del Río et al., 2007b, 2008). However, the original DFRC protocol cannot be used to assess the occurrence of naturally occurring acetates acylating the γ-OH of the lignin side chain because this degradation method uses acetate reagents that produces acetate derivatives; however, with a small modification of the original protocol by using propionylating reagents (so-called DFRC′) it was possible to obtain information about the occurrence of acetate groups naturally acylating the γ-OH (Ralph and Lu, 1998; del Río et al., 2007b). The analysis of the lignins from rice husks and straw by DFRC′ indicated that they were barely acylated with acetate groups (less than 0.5%), and confirmed that *p*-coumarate was the most important group acylating the γ-OH. Interestingly, significant amounts of tricin (T), as its acetate derivative, were also released from these lignins by DFRC (Figure 6), being more abundant in the lignin from rice straw than in the lignin from rice husks.

The results obtained from the DFRC and DFRC′ degradations of the MWLs isolated from rice husks and straw, including the molar yields of the released monomers (H, G, G_*ac*_, G_*pCA*_, S, S_*ac*_, S_*pCA*_, and T), as well as the percentages of naturally acetylated guaiacyl (%G_*ac*_) and syringyl (%S_*ac*_), and *p*-coumaroylated guaiacyl (%G_*pCA*_) and syringyl (%S_*pCA*_) lignin units, are shown in Table 5. The analyses confirmed that *p*-coumarate was the main group acylating the γ-OH of the side-chain in both lignins, and were preferentially attached to the S-lignin units (30.2% of total S-units, and only 0.5% of total G-units were *p*-coumaroylated in the lignin from rice husks, whereas 19.7% of total S-units, and only 1.2% of total G-units, were *p*-coumaroylated in the lignin from rice straw). The analysis indicated that acetates were also acylating the γ-OH in these lignins although at a very low level. In the lignin of rice husks, acylation with acetates represented only 0.5% of all G units and 0.1% of all S-units, whereas in the lignin of rice straw, acetates represented 0.4% of all G units, and 0.2% of all S-units. In both cases, acetylation occurred preferentially over G-lignin units. Finally, the DFRC confirmed the occurrence of significant amounts of tricin (T) incorporated into these lignins, being more abundant in the lignin of rice straw (accounting for 8.1% of all release lignin units), than in the lignin of rice husks (only 1.6% of all released lignin units), confirming the results obtained from 2D-NMR that indicated the occurrence of higher amounts of tricin incorporated into the lignin of rice straw than into the lignin of rice husks.

**TABLE 5:** Relative molar abundance of the normal γ-OH (H, G, S), γ-acetylated (G_*ac*_, S_*ac*_), and γ-*p*-coumaroylated (G_*pCA*_, S*_*p*_*_*cA*_) lignin units, and tricin (T), obtained from DFRC and DFRC′ degradation of the MWLs isolated from rice husks and straw.

	H	G	G_*ac*_	G_*pCA*_	S	S_*ac*_	S_*pCA*_	T^*a*^	%G_*ac*_^*b*^	%G_*pCA*_^*c*^	%S_*ac*_^*d*^	%S_*pCA*_^*e*^
Rice husks	10.2	83.6	0.5	0.4	3.6	0.1	1.6	1.6	0.6	0.5	1.9	30.2
Rice straw	12.5	73.5	0.4	0.9	10.0	0.2	2.5	8.1	0.5	1.2	1.5	19.7

*The percentages of the different acylated (acetylated and p-coumaroylated) lignin units are also shown.^a^T molar content referred as to the percentage of total lignin units (H + G + G_ac_ + G_pCA_ + S + S_ac_ + S_pCA_ = 100).^b^%G_ac_ is the percentage of acetylated G units (G_ac_) with respect to the total G units (G + G_ac_ + G_pCA_).^c%^G_pCA_ is the percentage of p-coumaroylated G units (G_pCA_) with respect to the total G units (G + G_ac_ + G_pCA_).^d^%S_ac_ is the percentage of acetylated S units (S_ac_) with respect to the total S units (S + S_ac_ + S_pCA_).^e^%S_pCA_ is the percentage of p-coumaroylated S units (S_pCA_) with respect to the total S units (S + S_ac_ + S_p__CA_).*

## Discussion

In this work, the detailed structural characteristics of the lignins of rice husks (with 22.5% lignin content) and rice straw (13.5% lignin content) were thoroughly analyzed by using an array of analytical techniques, including Py-GC/MS, 2D-NMR, and DFRC. The analyses indicated that both lignins were enriched in G-lignin units, and depleted in H- and S-lignin units, but with noticeable differences in the lignin composition between both tissues. The lignin of rice husks presented a H:G:S composition of 7:81:12 (S/G of 0.15) and was significantly more enriched in G-units than the lignin of rice straw, with a H:G:S composition of 5:71:24 (S/G of 0.34). Moreover, these lignins presented a higher content of G-lignin units, and consequently lower S/G ratios, than the lignins of similar tissues in other grasses. Hence, the lignin of rice husks presented a lower S/G ratio than the lignins of barley husks (S/G ∼ 0.5) (Rencoret et al., 2015), and more especially than the lignin of corn husks (S/G ∼ 2) (del Río et al., 2018). Likewise, the lignin of rice straw presented a lower S/G ratio than the lignins of wheat straw (S/G of 0.5) (del Río et al., 2012b), or sugarcane straw (S/G of 0.5) (del Río et al., 2015). The lignin composition greatly influenced the distribution of the different lignin inter-unit linkages in both lignins. As sinapyl alcohol (the precursor of the S-lignin units) presents two methoxyl groups in the aromatic ring, it can only form linkages at the β-position, being mostly β–*O*–4 alkyl aryl ether linkages, and β–β linkages to a lesser extent; on the contrary, coniferyl alcohol (the precursor of G-lignin units) has only one methoxyl group in the aromatic ring and presents a free position at C5 to form additional covalent linkages with another lignin unit, such as β–5 or 5–5 linkages, and producing a more condensed structure. Hence, the lignin from rice husks, that presented a higher abundance of G-lignin units, presented lower levels of β–*O*–4 alkyl aryl ether structures (65% of all measured linkages) and higher levels of condensed structures, particularly phenylcoumarans (23%), and dibenzodioxocins (5%). On the other hand, the lignin from rice straw, with a slightly higher S/G ratio, presented a higher level of β–*O*–4 alkyl aryl ether structures (78% of all measured linkages), and lower levels of phenylcoumarans (12%), and dibenzodioxocins (4%). The analyses, therefore, indicated that the lignins of rice husks and straw have a remarkably more condensed structure than the lignins from similar tissues in related grasses, and that the lignin from rice husks was significantly more condensed than the lignin from rice straw, being therefore more recalcitrant and less prone to chemical and biological degradation. Rice husks are the hard protecting coverings of rice grains, therefore, lignification of rice husks plays an important role in seed protection. The higher recalcitrance of the lignin in rice husks, together with their higher lignin content compared to rice straw, makes this lignin more difficult to degrade, which appears to play a role in protecting the seeds.

The lignins of rice husks and straw also presented significant amounts of *p*-hydroxycinnamates (*p*-coumarates and ferulates). Ferulates were found to be mostly attached to the carbohydrates, most likely to the arabinosyl residues of arabinoxylans, as occurred in other grasses, and are known to participate in radical coupling reactions with monolignols that cross-link the carbohydrates to the lignin network (Quideau and Ralph, 1997; Hatfield et al., 2017). On the other hand, *p*-coumarates were found partially acylating the γ-OH of the lignin side-chains (10 and 12% of all side-chains were *p*-coumaroylated in the lignins of rice husks and straw), and overwhelmingly over S-lignin units, as occurred in other grasses (Grabber et al., 1996; Lu and Ralph, 1999; Hatfield et al., 2008, 2009; Ralph, 2010; del Río et al., 2012a, 2015). The *p*-coumaroyl-CoA:monolignol transferases involved in the *p*-coumaroylation of the lignin have already been identified and characterized in some grasses and presented higher affinity toward sinapyl alcohol than toward coniferyl alcohol (Withers et al., 2012; Marita et al., 2014; Petrik et al., 2014). However, the role of lignin *p*-coumaroylation in grasses still remains unclear, although it has been suggested that *p*-coumarates may act as a radical transfer system to help in the radical coupling of sinapyl alcohol into the growing lignin polymer (Hatfield et al., 2008). On the other hand, the lignins from many plants, including grasses, also present acetate groups acylating the γ-OH of the lignin side-chain, and in some cases the acetylation degree occurs to a high extent, as occurred with sisal (78% acetylation level), kenaf (69%), or abaca (50%) (Ralph, 1996; del Río et al., 2007b, 2008). However, the lignins of rice husks and straw were barely acylated with acetate groups at the γ-OH; in the lignin from rice husks only 0.5% of all G-units and 0.1% of all S-units were acetylated, whereas in the lignin from rice straw, only 0.4% of all G-units, and 0.2% of all S-units were acetylated. Interestingly, and contrary to what occurs with *p*-coumarates, in both cases acetylation occurred preferentially over G-lignin units, a feature that has already been observed in the lignins from other grasses, as bamboo, wheat straw, sugarcane, or the pith of elephant grass (del Río et al., 2007b, 2012a, b, 2015). This fact seems to indicate that the acetyl-CoA:monolignol transferases involved in monolignol acetylation in grasses presents a higher affinity toward coniferyl alcohol than toward sinapyl alcohol, contrary to what occurs in most plants where acetylation usually takes place preferentially over S-lignin units (del Río et al., 2007b, 2008). The most important acylated monolignol in the lignins of rice husks and straw was, therefore, sinapyl *p*-coumarate, that accounted for 30.2% of the total S-units in rice husks, and for 19.7% of total S-units in rice straw. Sinapyl-*p*-coumarate has been shown to behave as a true lignin monomer participating in coupling and cross-coupling reactions during lignification, as it was demonstrated by the formation of γ-acylated β–β tetrahydrofuran structures produced from the β–β coupling of two γ-acylated monolignols, or by the cross-coupling of a γ-acylated and a normal γ-OH monolignol (Lu and Ralph, 2005; del Río et al., 2015). The occurrence of the characteristic signals from γ-*p*-coumaroylated β–β tetrahydrofuran structures (**C′**) in the HSQC spectrum of the lignin from rice straw clearly evidenced that sinapyl-*p*-coumarate behaves as a true lignin monomer in these rice tissues participating in coupling reactions during lignification.

Important amounts of the flavone tricin were also found incorporated into the lignin of rice straw (8.1% of total lignin units involved in β-ethers), and to a lesser extent, into the lignin of rice husks (1.6%). Tricin has mostly been found in the aerial parts of grasses, and therefore, it is likely that the higher amounts of tricin found in rice straw may be related to its potential role as UV-protecting agent, as already suggested (del Río et al., 2020). Tricin was the first phenolic compound from beyond the canonical monolignol biosynthetic pathway that was discovered to behave as a true lignin monomer participating in cross-coupling reactions with traditional monolignols during the lignification process and being integrally incorporated into the lignin polymer (del Río et al., 2012b; Lan et al., 2015). Further studies indicated that tricin was an important component of the lignin of all grasses (del Río et al., 2012b, 2015; Lan et al., 2015, 2016a,b) and that it was also incorporated into the lignins of other monocots, such as in coconut coir (from the Arecaceae), vanilla (from the Orchidaceae), or curaua (from the Bromeliaceae) (Rencoret et al., 2013; Lan et al., 2016b). Due to its particular structure, tricin cannot couple with another tricin molecule and its only possible mode of incorporation into the lignin polymer is through 4–*O*–β coupling with a monolignol, and consequently it must always be present at the starting end of a lignin chain; therefore, it seems that tricin may have a role as an initiation site for lignification in grasses (Lan et al., 2015, 2016a). The pathway for tricin biosynthesis in rice has recently been elucidated, and involved the condensation of *p*-coumaroyl-CoA and three malonyl-CoA units catalyzed by Chalcone Synthase (CHS) followed by isomerization by Chalcone Isomerase (CHI) to form the flavanone naringenin, which is then converted to the flavone apigenin by flavone synthase II (FNSII), and further sequential and consecutive hydroxylation and *O*-methylation steps leads to luteolin, chrysoeriol, selgin, and ultimately to tricin, and all the enzymes involved have been identified (Lam et al., 2015, 2017). The detailed knowledge of the biosynthetic pathway leading to tricin allowed producing genetically engineered rice with altered lignins that resulted in the incorporation of other flavonoids, such as the flavanone naringenin or the flavone apigenin into their structure, instead of tricin (Lam et al., 2017, 2019). Tricin can also occur in grasses in the form of extractives, such as free tricin, as *O*-glucosides, as flavonolignans, or as flavonolignan glucosides. A detailed quantitative study of the tricin content in several grasses demonstrated that the content of tricin incorporated into the lignin polymer was much higher than the content of extractable tricin (Lan et al., 2016b, 2019). In the case of rice straw, the content of tricin incorporated into the lignin amounted up to 980 mg/kg in comparison to only 195 mg/kg of extractable tricin (Lan et al., 2016b), which indicates that the lignin of rice straw could be an attractive and potential source for the extraction of this valuable compound. It is important to note that tricin presents multiple nutraceutical and pharmacological applications, and has a potential role as a chemopreventive and anticancer agent (Jian-Min and Ragai, 2010). The fact that tricin is linked to the lignin network exclusively by labile and easily cleaved β-ether bonds adds to the feasibility of considering rice straw lignin as a potential feedstock for obtaining valuable tricin.

In general terms, the detailed chemical and structural characteristics of the lignins of rice husks and rice straw provided in this work will be of great help for tailoring appropriate and efficient conversion technologies for these lignocellulosic materials as well as for developing lignin-based high value added products. Fractionation of rice by-products into their cell wall components (cellulose, hemicelluloses, and lignin) is a main step for their efficient utilization in integrated biorefineries and to maximize their value-added conversion into biofuels and chemicals. Various pretreatment techniques have been used for this purpose, including chemical (acid, alkaline, and oxidation) and thermochemical (steam explosion, autohydrolysis, and organosolv) methods, or combinations of them (Imman et al., 2015; Abraham et al., 2016; Singh and Dhepe, 2016; Wood et al., 2016; Wu et al., 2018; Swain et al., 2019; Takeda et al., 2019; Sharma et al., 2020). However, fractionation of cell wall components is greatly influenced by the lignin characteristics. As shown above, the lignins of rice husks and rice straw are highly recalcitrant and difficult to depolymerize, and it can be anticipated that severe and harsh conditions must be necessary to achieve delignification in order to access the carbohydrates. In particular, the data indicated that rice husks exhibited a much higher degree of recalcitrance compared to rice straw as a result of the higher lignin content and the higher degree of condensed linkages in this lignin. This explains the large differences observed in the degree of delignification between both materials reported in previous works (Wood et al., 2016; Wu et al., 2018); for example, and despite having a similar carbohydrate content, rice husks were found to be much less susceptible to saccharification by steam explosion, even under optimal pretreatments conditions, compared to rice straw (Wood et al., 2016). The results of this work indicate that the use of rice husks as raw material for biorefinery purposes would be difficult to achieve and that its potential exploitation will require severe conditions to cope with its recalcitrance. New developments in pretreatment technologies or breeding strategies to reduce the recalcitrance of rice husks have been suggested to address this problem (Wood et al., 2016).

On the other hand, the potential applications of the lignins extracted from the rice by-products will be determined by the lignin composition. Hence, the lignins from rice husks and rice straw are highly enriched in G-units and are suitable for producing phenols with unsubstituted C5 positions, which would provide the necessary reactivity to produce phenol formaldehyde (PF) resins, and therefore, could be appropriate for this type of application (de Menezes et al., 2017). Furthermore, these lignins can also produce significant amounts of non-methoxylated *p*-hydroxyphenyl units arising from the *p*-coumarate groups attached to the lignin side chains that could also provide reactivity for PF resin applications. However, it is important to note that the pretreatment procedures used to extract the lignins will also have a great impact on the structure, purity and physico-chemical properties of the recovered lignins and, hence, on their subsequent applications.

Finally, and perhaps more importantly, the results of this study indicate that these lignins also contain significant amounts of *p*-hydroxycinnamic acids, as well as the flavonoid tricin, which can be recovered as different side streams. The core lignin of these rice by-products is mostly composed of G-units, making them highly recalcitrant and difficult to degrade. However, as shown above, *p*-coumarates are esterified to the γ-OH of the lignin side chain, and can be easily released by mild alkaline treatment; likewise, tricin is linked exclusively by β-ether bonds, which can also be easily cleaved releasing this valuable flavonoid. Therefore, rice husks and rice straw may represent a promising source for these fine chemicals in the context of a lignocellulosic biorefinery.

## Data Availability Statement

The raw data supporting the conclusions of this article will be made available by the authors, without undue reservation.

## Author Contributions

MJR, GM, AG, and JR made the experimental work. JCR designed the work and wrote the article, with contributions from the rest of authors. All authors approved the version submitted.

## Conflict of Interest

The authors declare that the research was conducted in the absence of any commercial or financial relationships that could be construed as a potential conflict of interest.

## References

[B1] Abraham A. Mathew A. K. Sindhu R. Pandey A. Binod P. (2016). Potential of rice straw for bio-refining: an overview. *Bioresour. Technol.* 215 29–36. 10.1016/j.biortech.2016.04.011 27067674

[B2] Bhattacharyya P. Bhadyri D. Adak T. Munda S. Sataphaty B. S. Dash P. K. (2020). Characterization of rice straw from major cultivars for best alternative industrial uses to cutoff the menace of straw burning. *Ind. Crop Prod.* 143:111919. 10.1016/j.indcrop.2019.111919

[B3] Björkman A. (1956). Studies on finely divided wood. Part 1. Extraction of lignin with neutral solvents. *Sven. Papperstidn.* 59 477–485.

[B4] Bocchini P. Galletti G. C. Camarero S. Martínez A. T. (1997). Absolute quantitation of lignin pyrolysis products using an internal standard. *J. Chromatogr. A* 773 227–232. 10.1016/S0021-9673(97)00114-3

[B5] Browning B. L. (1967). *Methods of Wood Chemistry*, Vol. II. (New York, NY: Wiley-Interscience Publishers), 498.

[B6] Campbell M. M. Sederoff R. R. (1996). Variation in lignin content and composition. *Plant Physiol.* 110 3–13. 10.1104/pp.110.1.3 12226169 PMC157688

[B7] Chandrasekhar S. Satyanarayana K. G. Pramada P. N. Raghavan P. Gupta T. N. (2003). Processing, properties and applications of reactive silica from rice husk - An overview. *J. Mat. Sci.* 38 3159–3168. 10.1023/A:1025157114800

[B8] Dagnino E. P. Felissia F. E. Chamorro E. Area M. C. (2018). Studies on lignin extraction from rice husk by a soda-ethanol treatment: kinetics, separation and characterization products. *Chem. Eng. Res. Des.* 129 209–216. 10.1016/j.cherd.2017.10.026

[B9] Darwill A. McNeil M. Albersheim P. Delmer D. (1980). “The primary cell-walls of flowering plants,” in *The Biochemistry of Plants: A Comprehensive Treatise*, ed. Tolbert N. (New York, NY: Academic Press), 91–162. 10.1016/b978-0-12-675401-8.50009-9

[B10] del Río J. C. González-Vila F. J. Martín F. (1996). Thermally assisted hydrolysis and alkylation as a novel pyrolytic approach for the structural characterization of natural biopolymers and geomacromolecules. *Trends Anal. Chem.* 15 70–79. 10.1016/0165-9936(96)80763-1

[B11] del Río J. C. Gutiérrez A. Rodríguez I. M. Ibarra D. Martínez A. T. (2007a). Composition of non-woody plant lignins and cinnamic acids by Py-GC/MS, Py/TMAH and FT-IR. *J. Anal. Appl. Pyrol.* 79 39–46. 10.1016/j.jaap.2006.09.003

[B12] del Río J. C. Marques G. Rencoret J. Martínez A. T. Gutiérrez A. (2007b). Occurrence of naturally acetylated lignin units. *J. Agric. Food Chem.* 55 5461–5468. 10.1021/jf0705264 17552541

[B13] del Río J. C. Lino A. G. Colodette J. L. Lima C. F. Gutiérrez A. Martínez A. T. (2015). Differences in the chemical structure of the lignins from sugarcane bagasse and straw. *Biomass Bioenerg.* 81 322–338. 10.1016/j.biombioe.2015.07.006

[B14] del Río J. C. Prinsen P. Rencoret J. Nieto L. Jiménez-Barbero J. Ralph J. (2012a). Structural characterization of the lignin in the cortex and pith of elephant grass (*Pennisetum purpureum*) stems. *J. Agric. Food Chem.* 60 3619–3634. 10.1021/jf300099g 22414389

[B15] del Río J. C. Rencoret J. Prinsen P. Martínez Á. T. Ralph J. Gutiérrez A. (2012b). Structural characterization of wheat straw lignin as revealed by analytical pyrolysis, 2D-NMR, and reductive cleavage methods. *J. Agric. Food Chem.* 60 5922–5935. 10.1021/jf301002n 22607527

[B16] del Río J. C. Rencoret J. Gutiérrez A. Elder T. Kim H. Ralph J. (2020). Lignin monomers from beyond the canonical monolignol biosynthetic pathway – Another brick in the wall. *ACS Sustain. Chem. Eng.* 8 4997–5012. 10.1021/acssuschemeng.0c01109

[B17] del Río J. C. Rencoret J. Gutiérrez A. Kim H. Ralph J. (2017). Hydroxystilbenes are monomers in palm fruit endocarp lignins. *Plant Physiol.* 174 2072–2082. 10.1104/pp.17.00362 28588115 PMC5543948

[B18] del Río J. C. Rencoret J. Gutiérrez A. Kim H. Ralph J. (2018). Structural characterization of lignin from maize (*Zea mays* L.) fibers: evidence for diferuloylputrescine incorporated into the lignin polymer in maize kernels. *J. Agric. Food Chem.* 66 4402–4413. 10.1021/acs.jafc.8b00880 29665690

[B19] del Río J. C. Rencoret J. Marques G. Gutiérrez A. Ibarra D. Santos J. I. (2008). Highly acylated (acetylated and/or *p*-coumaroylated) native lignins from diverse herbaceous plants. *J. Agric. Food Chem.* 56 9525–9534. 10.1021/jf800806h 18823124

[B20] de Menezes F. F. Rencoret J. Nakanishi S. C. Nascimento V. M. Silva V. F. N. Gutiérrez A. (2017). Alkaline pretreatment severity leads to different lignin applications in sugar cane biorefineries. *ACS Sustain. Chem. Eng.* 5, 5702–5712. 10.1021/acssuschemeng.7b00265

[B21] Donaldson L. A. (2001). Lignification and lignin topochemistry – an ultrastructure view. *Phytochemistry* 57 859–873. 10.1016/S00319422(01)00049-8 11423137

[B22] FAOSTAT (2020). *Food and Agriculture Organization of the United Nations.* Available online at: http://faostat3.fao.org (accessed November 4, 2020).

[B23] Gao Y. Guo X. Liu Y. Fang Z. Zhang R. You L. (2018). A full utilization of rice husk to evaluate phytochemical bioactivities and prepare cellulose nanocrystals. *Sci. Rep.* 8:10482. 10.1038/s41598-018-27635-3 29992951 PMC6041302

[B24] Gou G. Wei W. Jiang M. Zhang S. Tingju L. Xie X. (2018). “Environmentally friendly method for the separation of cellulose from steam-exploded rice straw and its high-value applications,” in *Pulp and Paper Processing*, ed. Kazi S. N. (London: IntechOpen), 133–154. 10.5772/intechopen.79014

[B25] Grabber J. H. Quideau S. Ralph J. (1996). p-Coumaroylated syringyl units in maize lignin: implications for β-ether cleavage by thioacidolysis. *Phytochemistry* 43 1189–1194. 10.1016/S0031-9422(96)00431-1

[B26] Hatfield R. Ralph J. Grabber J. H. (2008). A potential role for sinapyl *p*-coumarate as a radical transfer mechanism in grass lignin formation. *Planta* 228 919–928. 10.1007/s00425-008-0791-4 18654797

[B27] Hatfield R. D. Marita J. M. Frost K. Grabber J. Ralph J. Lu F. (2009). Grass lignin acylation: *p*-coumaroyl transferase activity and cell wall characteristics of C3 and C4 grasses. *Planta* 229 1253–1267. 10.1007/s00425-009-0900-z 19288269

[B28] Hatfield R. D. Rancour D. M. Marita J. M. (2017). Grass cell walls: a story of cross-linking. *Front. Plant Sci.* 7:2056. 10.3389/fpls.2016.02056 28149301 PMC5241289

[B29] Imman S. Arnthong J. Burapatana V. Champreda V. Laosiripojana N. (2015). Fractionation of rice straw by a single-step solvothermal process: effects of solvents, acid promoters, and microwave treatment. *Renew. Energy* 83 663–673. 10.1016/j.renene.2015.04.062

[B30] Jian-Min Z. Ragai I. (2010). Tricin—a potential multifunctional nutraceutical. *Phytochem. Rev.* 9 413–424. 10.1007/s11101-009-9161-5

[B31] Kalita E. Narth B. K. Deb P. Agan F. Islam M. R. Saikia K. (2015). High quality fluorescent cellulose nanofibers from endemic rice husk: isolation and characterization. *Carbohydr. Polym.* 122 308–313. 10.1016/j.carbpol.2014.12.075 25817673

[B32] Kim H. Ralph J. Akiyama T. (2008). Solution-state 2D NMR of ball-milled plant cell-wall gels in DMSO-*d*_6_. *Bioenergy Res.* 1 56–66. 10.1007/s12155-008-9004-z PMC4070321 20090974

[B33] Kumar A. K. Parikh B. S. Pravakar M. (2016). Natural deep eutectic solvent mediated pretreatment of rice straw: bioanalytical characterization of lignin extract and enzymatic hydrolysis of pretreated biomass residue. *Environ. Sci. Pollut. Res.* 23 9265–9275. 10.1007/s11356-015-4780-4 26032452

[B34] Kumar M. Upadhyay S. N. Mishra P. K. (2019). A comparative study of thermochemical characteristics of lignocellulosic biomasses. *Bioresour. Technol. Rep.* 8:100186. 10.1016/j.biteb.2019.100186

[B35] Lam P. Y. Liu H. Lo C. (2015). Completion of tricin biosynthesis pathway in rice: cytochrome P450 75B4 is a unique chrysoeriol 5’-hydroxylase. *Plant Physiol.* 168 1527–1536. 10.1104/pp.15.00566 26082402 PMC4528758

[B36] Lam P. Y. Tobimatsu Y. Matsumoto N. Suzuki S. Lan W. Takeda Y. (2019). OsCAldOMT1 is a bifunctional *O*-methyltransferase involved in the biosynthesis of tricin-lignins in rice cell walls. *Sci. Rep.* 9:11597. 10.1038/s41598-019-47957-0 31406182 PMC6690965

[B37] Lam P. Y. Tobimatsu Y. Takeda Y. Suzuki S. Yamamura M. Umezawa T. (2017). Disrupting flavone synthase II alters lignin and improves biomass digestibility. *Plant Physiol.* 174 972–985. 10.1104/pp.16.01973 28385728 PMC5462022

[B38] Lan W. Lu F. Regner M. Zhu Y. Rencoret J. Ralph S. A. (2015). Tricin, a flavonoid monomer in monocot lignification. *Plant Physiol.* 167 1284–1295. 10.1104/pp.114.253757 25667313 PMC4378158

[B39] Lan W. Morreel K. Lu F. Rencoret J. del Río J. C. Vooren W. (2016a). Maize tricin-oligolignol metabolites and their implications for monocot lignification. *Plant Physiol.* 171 810–820. 10.1104/pp.16.02012 27208246 PMC4902589

[B40] Lan W. Rencoret J. Lu F. Karlen S. D. Smith B. G. Harris P. J. (2016b). Tricin-lignins: occurrence and quantitation of tricin in relation to phylogeny. *Plant J.* 88 1046–1057. 10.1111/tpj.13315 27553717

[B41] Lan W. Rencoret J. del Río J. C. Ralph J. (2019). “Tricin in grass lignin: biosynthesis, characterization, and quantitation,” in *Lignin: Biosynthesis, Functions and Economic Significance*, eds Lu F. Yue F. (New York, NY: Nova Science Publishers, Inc), 51–78.

[B42] Lourenço A. Rencoret J. Chematova C. Gominho J. Gutiérrez A. del Río J. C. (2016). Lignin composition and structure differs between xylem, phloem and phellem in *Quercus suber* L. *Front. Plant Sci.* 7:1612. 10.3389/fpls.2016.01612 27833631 PMC5081372

[B43] Lu F. Ralph J. (1997a). Derivatization followed by reductive cleavage (DFRC method), a new method for lignin analysis: protocol for analysis of DFRC monomers. *J. Agric. Food Chem.* 45 2590–2592. 10.1021/jf970258h

[B44] Lu F. Ralph J. (1997b). The DFRC method for lignin analysis. 1. New method for β-aryl ether cleavage: lignin model studies. *J. Agric. Food Chem.* 45 4655–4660. 10.1021/jf970539p

[B45] Lu F. Ralph J. (1998). The DFRC method for lignin analysis. 2. Monomers from isolated lignin. *J. Agric. Food Chem.* 46 547–552. 10.1021/jf970676m 10554275

[B46] Lu F. Ralph J. (1999). Detection and determination of *p*-coumaroylated units in lignins. *J. Agric Food Chem.* 47 1988–1992. 10.1021/jf981140j 10552483

[B47] Lu F. Ralph J. (2005). Novel β–β Structures in lignins incorporating acylated monolignols. *Appita* 233–237.

[B48] Lu P. Hsieh Y. L. (2012). Preparation and characterization of cellulose nanocrystals from rice straw. *Carbohydr. Polym.* 87 564–573. 10.1016/j.carbpol.2011.08.022 34663005

[B49] Marita J. M. Hatfield R. D. Rancour D. M. Frost K. E. (2014). Identification and suppression of the *p*-coumaroyl CoA:hydroxycinnamyl alcohol transferase in *Zea mays* L. *Plant J.* 78 850–864. 10.1111/tpj.12510 24654730 PMC4282748

[B50] Petrik D. Karlen S. D. Cass C. Padmakshan D. Lu F. Liu S. (2014). *p*-Coumaroyl-CoA:monolignol transferase (PMT) acts specifically in the lignin biosynthetic pathway in *Brachypodium distachyon*. *Plant J.* 77 713–726. 10.1111/tpj.12420 24372757 PMC4282527

[B51] Quideau S. Ralph J. (1997). Lignin-ferulate cross-links in grasses. Part 4. Incorporation of 5–5-coupled diferulate into lignin. *J. Chem. Soc. Perkin Trans.* 1 2351–2358. 10.1039/A701808h

[B52] Ralph J. (1996). An unusual lignin from kenaf. *J. Nat. Prod.* 59 341–342. 10.1021/np960143s

[B53] Ralph J. (2010). Hydroxycinnamates in lignification. *Phytochem. Rev.* 9 65–83. 10.1007/s11101-009-9141-9

[B54] Ralph J. Hatfield R. D. (1991). Pyrolysis-GC/MS characterization of forage materials. *J. Agric. Food Chem.* 39 1426–1437. 10.1021/jf00008a014

[B55] Ralph J. Lu F. (1998). The DRC method for lignin analysis. 6. A simple modification for identifying natural acetates in lignin. *J. Agric. Food Chem.* 46 4616–4619. 10.1021/jf980680d

[B56] Ralph J. Lundquist J. Brunow G. Lu F. Kim H. Schatz P. F. (2004). Lignins: natural polymers from oxidative coupling of 4-hydroxyphenylpropanoids. *Phytochem. Rev.* 3 29–60. 10.1023/b:phyt.0000047809.65444.a4

[B57] Ralph S. A. Ralph J. Landucci L. (2009). *NMR Database of lignin and Cell Wall Model Compounds.* Available online at: https://www.glbrc.org/databases_and_software/nmrdatabase/NMR_DataBase_2009_Complete.pdf (accessed April, 2020).

[B58] Rencoret J. Gutiérrez A. Nieto L. Jiménez-Barbero J. Faulds C. B. Kim H. (2011). Lignin composition and structure in young versus adult *Eucalyptus globulus* plants. *Plant Physiol.* 155 667–682. 10.1104/pp.110.167254 21098672 PMC3032458

[B59] Rencoret J. Kim H. Anderson B. E. Gutiérrez A. Ralph J. del Río J. C. (2018). Variability in lignin composition and structure in cell walls of different parts of macaúba (*Acrocomia aculeata*) palm fruit. *J. Agric. Food Chem.* 66 138–153. 10.1021/acs.jafc.7b04638 29241332

[B60] Rencoret J. Marques G. Gutiérrez A. Nieto L. Santos J. I. Jiménez-Barbero J. (2009). HSQC-NMR analysis of lignin in woody (*Eucalyptus globulus* and *Picea abies*) and non-woody (*Agave sisalana*) ball-milled plant materials at the gel state. *Holzforschung* 63 691–698. 10.1515/HF.2009.070

[B61] Rencoret J. Prinsen P. Gutiérrez A. Martínez A. T. del Río J. C. (2015). Isolation and structural characterization of the milled wood lignin, dioxane lignin, and cellulolytic lignin preparations from brewer’s spent grain. *J. Agric. Food Chem.* 63 603–613. 10.1021/jf505808c 25520237

[B62] Rencoret J. Ralph J. Marques G. Gutiérrez A. Martínez A. T. del Río J. C. (2013). Structural characterization of lignin isolated from coconut (*Cocos nucifera*) coir fibers. *J. Agric. Food Chem.* 61 2434–2445. 10.1021/jf304686x 23398235

[B63] Salanti A. Zoia L. Orlandi M. Zanini F. Elegir G. (2010). Structural characterization and antioxidant activity evaluation of lignin from rice husk. *J. Agric. Food Chem.* 58 10049–10055. 10.1021/jf102188k 20735133

[B64] Sharma A. Singh G. Arya S. K. (2020). Biofuel from rice straw. *J. Clean. Prod.* 277:124101. 10.1016/j.jclepro.2020.124101

[B65] Singh S. K. Dhepe P. L. (2016). Isolation of lignin by organosolv process from different varieties of rice husk: understanding their physical and chemical properties. *Bioresour. Technol.* 221 310–317. 10.1016/j.biortech.2016.09.042 27648850

[B66] Swain M. R. Singh A. Sharma A. K. Tuli D. K. (2019). “Bioethanol production from rice- and wheat straw: an overview,” in *Bioethanol Production from Food Crops: Sustainable Sources, Interventions and Challenges*, eds Ray R. C. Ramachandran S. (London: Academic Press), 213–231. 10.1016/B978-0-12-813766-6.00011-4

[B67] Takeda Y. Tobimatsu Y. Yamamura M. Takano T. Sakamoto M. Umezawa T. (2019). Comparative evaluations of lignocellulose reactivity and usability in transgenic rice plants with altered lignin composition. *J. Wood Sci.* 65:6. 10.1186/s10086-019-1784-6

[B68] Tappi Standard Test Methods 2004-2005 (2004). *Tappi Test Methods.* Atlanta, GA: Tappi Press.

[B69] Vanholme R. De Meester B. Ralph J. Boerjan W. (2019). Lignin biosynthesis and its integration into metabolism. *Curr. Opin. Biotechnol.* 56 230–239. 10.1016/j.copbio.2019.02.018 30913460

[B70] Vanholme R. Demedts B. Morreel K. Ralph J. Boerjan W. (2010). Lignin biosynthesis and structure. *Plant Physiol.* 152 895–905. 10.1104/pp.110.155119 20472751 PMC2899938

[B71] Vermerris W. Boon J. J. (2001). Tissue specific patterns of lignification are disturbed in the *brown midrib2* mutant of maize (*Zea mays* L.). *J. Agric. Food Chem.* 49 721–728. 10.1021/jf000740r 11262019

[B72] Withers S. Lu F. Kim H. Zhu Y. Ralph J. Wilkerson C. G. (2012). Identification of a grass-specific enzyme that acylates monolignols with *p*-coumarate. *J. Biol. Chem.* 287 8347–8355. 10.1074/jbc.M111.284497 22267741 PMC3318722

[B73] Wood I. P. Cao H.-G. Tran L. Cook N. Ryden P. Wilson D. R. (2016). Comparison of saccharification and fermentation of steam exploded rice straw and rice husk. *Biotechnol. Biofuels* 9:193. 10.1186/s13068-016-0599-6 27602056 PMC5011935

[B74] Wu J. Collins S. R. A. Elliston A. Wellner N. Dicks J. Roberts I. N. (2018). Release of cell wall phenolic esters during hydrothermal pretreatment of rice husks and rice straw. *Biotechnol. Biofuels* 11:162. 10.1186/s13068-018-1157-1 29991964 PMC5994648

[B75] Yeframova S. Zharmenov A. Sukharnikov Y. Bunchuk L. Kablanbekov A. Anarbekov K. (2019). Rice husk hydrolytic lignin transformation in carbonization process. *Molecules* 24:3075. 10.3390/molecules24173075 31450574 PMC6749279

